# Logistic challenges in implementing a multispecialty robotic surgery program at university hospitals

**DOI:** 10.1016/j.clinsp.2026.101025

**Published:** 2026-07-16

**Authors:** Antonio Jose Rodrigues Pereira, Ricardo Zugaib Abdalla, Ivan Cecconello, William Carlos Nahas, Evelinda Marramon Trindade, Ana Maria Malik

**Affiliations:** a Business Administration São Paulo School of Business Administration of Getulio Vargas Foundation, São Paulo, SP, Brazil; b Hospital das Clínicas da Faculdade de Medicina da Universidade de São Paulo (HCFMUSP), São Paulo, SP, Brazil; c Gastroenterology Department, Hospital das Clínicas da Faculdade de Medicina da Universidade de São Paulo (HCFMUSP), São Paulo, SP, Brazil; d Urology Department, Hospital das Clínicas da Faculdade de Medicina da Universidade de São Paulo (HCFMUSP), São Paulo, SP, Brazil; e Medicine (Preventive Medicine) from the Universidade de São Paulo, São Paulo, SP, Brazil

**Keywords:** Robotic surgery, Multispecialty program, Public hospital, Logistical challenges, Technology adoption, Healthcare management, Organizational change, Implementation science, Surgical innovation, Operational efficiency, Training, Cost analysis, Stakeholder engagement, Infrastructure, Patient outcomes

## Abstract

**Introduction:**

The integration of innovative, high-cost technologies like robotic surgery presents significant managerial and logistical challenges within public university hospitals. While previous research often highlights clinical outcomes, pragmatic studies on the administrative and organizational complexities of implementation are scarce. This manuscript aims to address this gap by analyzing the multifaceted logistical issues encountered during the establishment of a multispecialty robotic surgery program.

**Objective:**

This study's primary objective is to evaluate the managerial and logistical aspects of adopting a surgical robot in a public hospital, focusing on the administrative processes, institutional adjustments, and systemic challenges that impact patient care and institutional sustainability. The authors sought to identify key lessons learned from this experience and enumerate the institutional knowledge gained during the integration of this technology.

**Materials and methods:**

This study utilized a mixed-methods approach, combining an analysis of institutional data and semi-structured interviews with managers and professionals involved in the decision-making and adoption process. Data collected included demographic details, surgical times, and postoperative outcomes, which served as management indicators. The study's framework was informed by organizational and diffusion of innovation theories to reconstruct the history of the decision-making process.

**Results:**

The implementation of robotic surgery required substantial adjustments to hospital infrastructure, work processes, and personnel training. Key logistical difficulties documented included dependency on a single supplier, delays in importing materials, high costs without market competition, and extensive training requirements. While robotic surgery demonstrated clinical advantages, such as lower complication rates (4.2%vs. 5.2%) and reduced blood loss (266.2 mL vs. 598.2 mL), its successful adoption was contingent on detailed planning and effective management to minimize these logistical challenges and optimize benefits. The study also revealed a protracted learning curve for the surgical teams, although operative times showed a decreasing trend with increased experience.

**Conclusion:**

The successful implementation of robotic surgery in a public teaching hospital requires a dedicated focus on logistical planning, stakeholder engagement, and adaptive management. Preemptive modeling of costs, diversified supplier relationships, and phased training are essential for sustainability. These findings provide valuable guidance for similar institutions aiming to integrate disruptive surgical technologies.

## Highlights

• Rigorous analysis of logistical challenges in multispecialty robotic surgery.
• Used implementation science frameworks CFIR, TDF, and NoMAD to guide evaluation.
• Quantified 12‒18 month surgeon learning curve with SMEQ and LED data analysis.
• Robotic surgery showed longer times but better recovery, less blood loss.
• Emphasizes the need for diversified procurement, financial modeling, and partnerships.

## Introduction

Healthcare technology adoption in public university hospitals presents unique implementation challenges that extend far beyond clinical efficacy. High-cost innovations such as robotic surgery require intricate planning across financial, supply chain, workflow, and training domains, often under conditions of bureaucratic complexity and resource constraint.^1^^,^^2^ While evidence of the clinical benefits of robotic surgery is robust, there is a critical gap in the literature regarding the real-world logistical processes and barriers in publicly funded institutions, particularly in emerging economies.^3^^,^^4^ These challenges are compounded by public procurement regulations, budget uncertainties, and the dual mission of patient care and professional education.^5^^,^^6^

This study systematically analyzes the practical hurdles, strategies, and institutional lessons of a robotic surgery program at a major Brazilian Public University Hospital.^7^ Using the Consolidated Framework for Implementation Research (CFIR) to structure qualitative data collection and analysis, the authors document the specific barriers, adaptive strategies, and lessons learned throughout the implementation process.^8^, ^9^, ^10^, ^11^, ^12^ Clinical trial data are presented as contextual background to demonstrate the operational environment in which these logistical challenges occurred.

## Materials and methods

This study utilized exploratory and descriptive methods, incorporating a case study on the implementation of robotic surgery within a funded oncology program. The original trial, upon which the data is based, was a randomized controlled block study, approved by the Hospital Board and Ethical Committee (CEP-CAPPESQ n°473,694). The study was registered in the National Clinical Trial Registry (NCT02292914).

This research was supported by agreement N#771,232/2012 between the Ministry of Health’s Program for the Development of the Health Industrial Complex (PROCIS/DECIIS-MS) and the São Paulo State Department of Health. The project aimed to address the Ministry of Health’s inquiry regarding the usability of robotic surgery in total public assistance at the Cancer Institute of São Paulo State (ICESP), which is part of HCFMUSP. The study protocol was executed in accordance with the Methodological Guidelines and Economic Evaluation Framework of the Ministry of Health, including detailed operational micro-costing.^13^^,^^14^

The patient cohort consisted of individuals diagnosed with cancer, treated between 2014 and 2021, at ICESP, who were eligible for open surgery, video endoscopic, and robotic-assisted surgical approaches. All patients were additional patients from the public system, with no interference in the routine programmed surgeries.

The study included 08 subprojects across five surgical specialties:

• Digestive System Surgery: Transthoracic esophagectomy, subtotal gastrectomy with D2 lymphadenectomy, partial pancreatectomy, and proctosigmoidectomy with lymphadenectomy.
• Urologic Surgery: Prostatectomy.
• Gynecological Surgery: Hysterectomy with pelvic and para-aortic lymphadenectomy.
• Head and Neck Surgery: Transoral resection of malignant tumors of the mouth and oropharyngolarynx.
• Thoracic Surgery: Pulmonary lobectomies.

All participants provided informed consent. Patient data were collected and recorded in the Instituto do Câncer do Estado de São Paulo database.

### Data collection and theoretical framework

A mixed-methods approach was taken by combining institutional records with qualitative data to reconstruct the decision-making process. Specifically, this study incorporated a post-hoc analysis of original randomized trial data alongside a structured qualitative evaluation. For the clinical data, details were summarized as previously described. Regarding the qualitative component, semi-structured interviews were conducted with 14 key stakeholders, including hospital administrators (n = 3), specialty surgical leads (n = 4) representing five different specialties, procurement and supply chain staff (n = 2), nursing managers (n = 2), robotics program trainers (n = 2), and the biomedical engineering lead (n = 1).

The study utilized the Consolidated Framework for Implementation Research (CFIR), the Theoretical Domains Framework (TDF), and the Normalization Process Theory Measure (NoMAD) as guiding frameworks for the evaluation.^15^, ^16^, ^17^ The interview guide, available in the Supplementary Material, addressed all major domains of the CFIR, with particular focus on intervention characteristics, the inner setting, process, and stakeholder attributes. Interviews were coded through thematic analysis aligned with CFIR constructs, with themes iteratively reviewed and discussed among three investigators until consensus was reached. The data collection process included semi-structured interviews conducted with managers and professionals who were directly involved in the decision-making process and the adoption of the new surgical technology.

Data saturation was achieved after the twelfth interview, as confirmed by two subsequent interviews that revealed no additional themes. Direct quotations from stakeholders are presented to highlight each identified theme.

To analyze the adoption of robotic surgery, several organizational theories and innovation diffusion instruments were applied. Each theoretical perspective contributed to a comprehensive understanding of the factors influencing technology adoption and the integration process within the healthcare institution.

A broad range of variables and indicators were tracked throughout the study: these included intraoperative complications, estimated blood loss, the number of lymph nodes removed, length of postoperative hospital stay, days spent in the ICU, patient quality of life, use of opioid painkillers, pain scores, and rates of hospital readmission. Researchers recorded metrics such as total surgery time, time for docking the robotic system, and time spent at the surgical console.

Logistical/Ergonomic Assessment: Surgeons provided feedback using the Subjective Mental Effort Questionnaire (SMEQ) and the Local Experienced Discomfort (LED) Scale, which assessed ergonomics, personal safety, and their overall satisfaction with the procedures.^18^^,^^19^ While the study monitored procedure-specific costs and overall expenditures, it did not include costs associated with training, purchasing, or maintaining equipment, nor any indirect costs.

### Statistical analysis

A post-hoc statistical analysis was conducted on the data from the original trial. The analysis focused on management indicators, including length of hospital stay, adverse events, and resource utilization. Different statistical tests were applied depending on the distribution of the data, with statistical significance set at a p-value of 0.05 (5%).

## Results

A randomized cohort of 783 patients undergoing eight cancer surgeries (including procedures in gastroenterology, gynecology, head and neck, urology, and pulmonology) was evaluated. The patients were divided into three groups: Robotic (n = 421), Videosurgery (n = 173), and Conventional-Open (n = 189). The analysis of institutional data and interviews revealed findings across three major domains: organizational impact, clinical outcomes, and resource utilization.

The absolute distribution of costs for control procedures (mostly video endoscopy, except prostatectomies and gastrectomies, which were open surgery) and the incremental mean differences for robotic procedures, as well as deviations in length of hospital stay and total costs in the postoperative period, are represented in Table 1.

**Table 1:** Summary table of hospitalization and cost data by surgery type (Approximate monetary values expressed in contemporaneous U.S. dollars).

**Type of Surgery**	**Robot (n)**	**Difference Hospital Stay (Days, Robot vs. Control)**	**Incremental Cost (Robot vs. Control)**	**Control Group (Type/n)**	**Mean Hospital Stay (Control, Days)**	**Mean Cost (Control, R$)**
Esophagectomy	21	−3.6	± US$ 2,933.68 ± US$ 795.08	Videolap/22	20.3 ± 7.4	US$ 9,53.91 ± US$2729.96
Gastrectomy	20	−0.2	+US$ 2737.92 ± US$328.77	Open/24	11.5 ± 4.1	US$ 4169.93 ±US$ 1033.23
Rectal surgeries	24	−1.1	+US$1428.80 ±US$336.33	Videolap/24	10.0 ± 2.5	US$4673.72 ±US$990.34
Pancreatectomy	29	7.8 ± 1.2	US$4468.79 ±US$707.78	‒	‒	‒
Hysterectomy	43	+0.1	+US$319.54 ±US$26.02	Videolap/42	3.3 ± 1.3	US$4118.01 ±US$1879.83
Head & Neck	36	−2.5	+US$305.06 ±US$286.95	Video/55	7.3 ± 5.4	US$3451.93 ±US$1658.86
Prostatectomy	169	−0.7	+US$1806.27 ±US$618.05	Open/167	3.7 ± 0.9	US$1680.76 ± US$357.03
Pulmonary lobectomy	43	−0.3	-US$78.64 ±US$486.61	Videolap/40	6.0 ± 3.1	US$6376.17 ±US$394.88

There were no major complications or mortality related to the surgeries in any of the studied groups. Quality of life and functional scores tended to be better up to 12-months and were similar in extended follow-up.

**Fig. 1:**
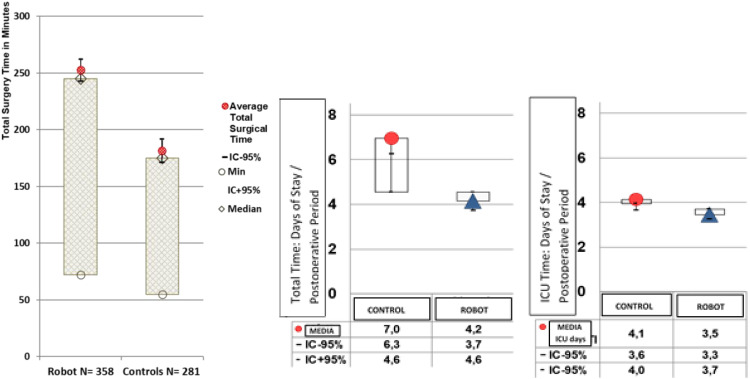
Absolute distribution of means and variability, 95% Confidence Intervals, times in minutes of use of the operating room and days of stay in the total postoperative period and Intensive Care (ICU) by control or robotic surgery group.

Fig. 1 shows the study main results, including absolute means, variability, 95% Confidence Intervals, operating room usage times, and postoperative and ICU stay durations for both control and robotic surgery groups.

The mean total operative time for robotic surgery was significantly longer than that for control surgeries (252 ± 94.2 min vs. 180.2 ± 87.8 min; p < 0.001). Despite this increased time, there was a notable reduction in postoperative hospital stay across all subprojects, as detailed in the supplementary material. The variation in operative times over the years showed a decreasing trend, correlating strongly with the surgical teams' experience.

Complications were lower in the robotic surgery group compared to the control group (4.2%vs. 5.2%; p = 0.57), with no major complications reported in either group. Robotic surgeries also resulted in significantly lower estimated median/mean blood loss (266.2 ± 292.9 mL vs. 598.2 ± 539.9 mL; p < 0.001) and reduced the need for blood transfusions. There were no surgical deaths in either group.

The length of postoperative recovery was shorter for robotic surgery patients, both in the ward and the ICU.

**Fig. 2:**
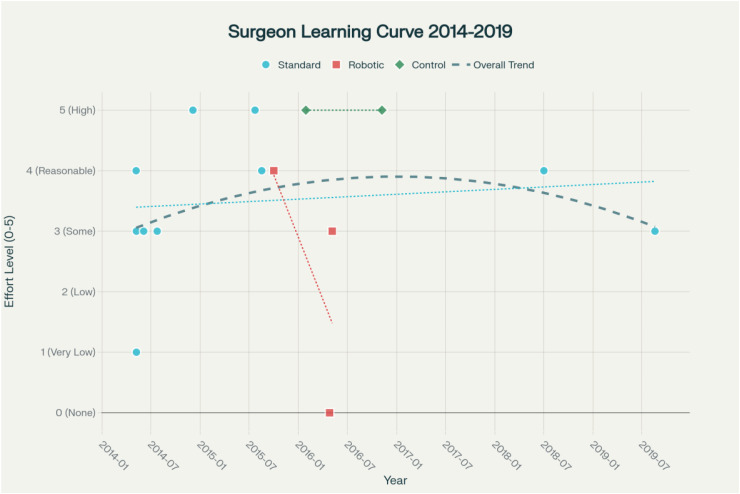
Legumina curve progression in robotic surgery implementation: SMEQ effort levels over time (2014‒2019 ‒ ICESP-HCFMUSP).

**Fig. 3:**
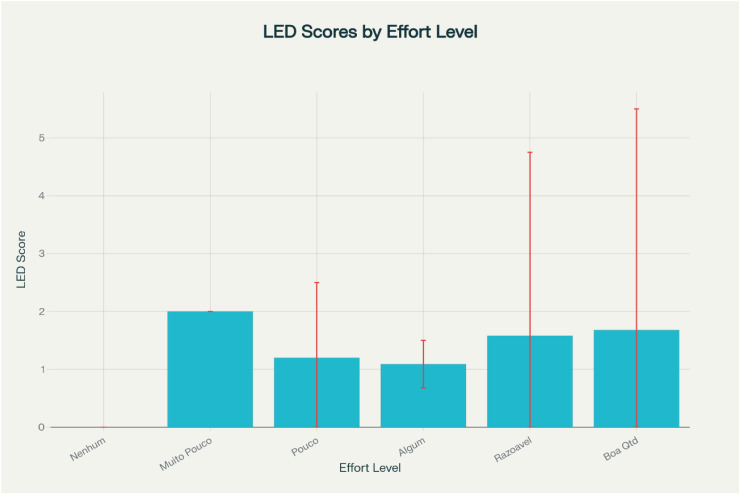
Correlation between subjective mental effort (SMEQ) and local experienced discomfort (LED) Scores.

**Fig. 4:**
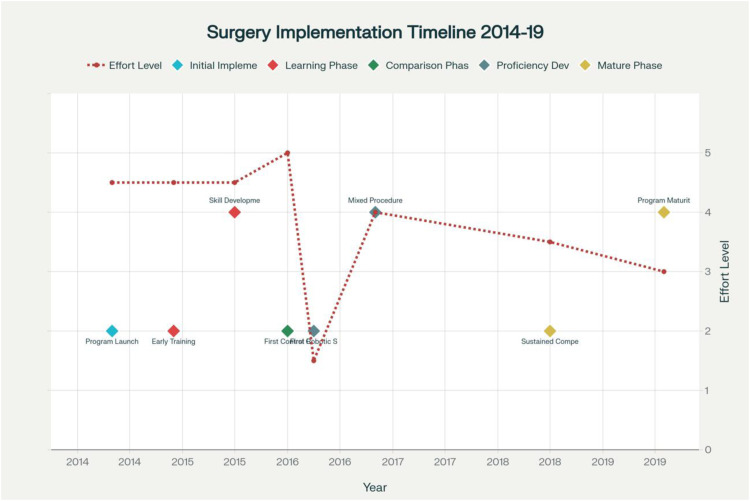
Robotic surgery program implementation timeline and learning curve phases (2014‒2019).

Over the study period, surgeons' increased experience improved their ability to handle complex cases efficiently. This improvement was reflected in the 320 responses to the Subjective Mental Effort Questionnaire (SMEQ) and Local Experienced Discomfort (LED) Scale. Fig. 2 demonstrates the quantified learning curve progression over 5-years, with effort levels declining from 4‒5 (high) in 2014 to 0‒3 (low) by 2019. The correlation between SMEQ and LED measurements (Fig. 3) validates the assessment methodology, while the implementation timeline (Fig. 4) maps key logistical challenges to specific program phases. Initial procedures required ‘*Boa Quantidade de Esforço*’ (High Effort) or ‘*Quantidade Razoável de Esforço*’ (Reasonable Effort), with scores ranging from 3‒7 on complexity scales. Over 12‒18 months, most surgeons achieved ‘*Muito Pouco Esforço*’ (Very Little Effort) or ‘*Nenhum Esforço*’ (No Effort) levels, with scores consistently at 0‒1 (Fig. 2). The proportion of responses indicated that gynecological surgeries were more challenging, whereas head and neck surgeries were less difficult.

## Discussion

The introduction of robotic surgery brought about substantial changes in hospital infrastructure, operational processes, and personnel training. Among the most significant challenges were the reliance on a single supplier, frequent delays in the importation of materials, elevated costs in the absence of market competition, and the need for comprehensive training of staff. Structural modifications became necessary, notably the doubling of operating room capacity, alongside considerable investments to support initial and ongoing training programs.

Analysis of operative times revealed a clear trend of reduction over the years, strongly associated with the enhanced experience of surgical teams and confirming the existence of an extended learning curve. Learning curve analysis using validated SMEQ and LED instruments demonstrated significant variation in surgeon adaptation rates across 15 procedures from 12 surgeons over 5-years. Early procedures required substantial effort levels (mean effort score 4.2 ± 0.8), with LED scores indicating moderate to high physical demands (peak scores up to 7.0). Over 12‒18 months, most surgeons achieved minimal effort levels (mean score 1.3 ± 0.5), with 53.3% of procedures initially requiring high effort (≥ 4) compared to only 13.3% achieving low effort (≤ 2) in the early phase.

“We greatly underestimated the time and cases needed for surgeons to be comfortable. Initial block times were rarely sufficient”, (Robotics Trainer, Interview #10). This was confirmed by learning curve data showing an average of 10 procedures needed to achieve proficiency levels. Feedback collected from surgeons was predominantly positive, indicating that, with increased familiarity, improvements in ergonomics and overall satisfaction were evident.

Despite the logistical obstacles, clinical and efficiency outcomes demonstrated the benefits of robotic surgery for patient care. Notably, although robotic procedures were associated with longer operative times, they consistently resulted in shorter postoperative hospital stays and reduced durations in intensive care units when compared to traditional approaches. For instance, patients undergoing esophagectomies experienced a reduction in hospitalization by 3.6-days, while those undergoing prostatectomies saw a decrease of 0.7 days relative to open procedures.

Further, the robotic group exhibited favorable clinical results, including a lower rate of complications (4.2%) compared to the control group (5.2%), though this difference did not achieve statistical significance. Blood loss during surgery was also significantly reduced, with the median/mean volume for robotic interventions at 266.2 ± 292.9 mL, as opposed to 598.2 ± 539.9 mL in the control group, with this difference being statistically significant (p < 0.001). Importantly, no major complications or surgical mortalities were recorded in any of the study groups.

The assessment of resource utilization highlighted a notable trade-off: while the mean operative time for robotic surgery was substantially longer (252 ± 94.2 min) than for conventional surgeries (180.2 ± 87.8 min, p < 0.001), the reduced length of hospital stays translated into efficiency gains. For example, pulmonary lobectomies performed robotically resulted in a cost saving of R$ 420.60 (approximately US$80.00), whereas most other procedures entailed higher operative costs, which were subsequently balanced by the accelerated recovery observed in patients.

The findings of this study confirm a crucial dichotomy in the diffusion of high-cost medical technology, specifically robotic surgery, within the context of a large, publicly funded university hospital. While the clinical results, notably reduced blood loss and shorter postoperative hospital stay, affirm the clinical advantages widely reported in the literature, the primary contribution of this work lies in systematically evaluating and articulating the profound managerial and logistical challenges inherent to such implementation in the public sector.

The implementation challenges documented here are not unique to robotic surgery but are often magnified within publicly funded health systems due to rigid procurement processes, constrained budgets, and reliance on centralized decision-making.

• Monopoly and Financial Strain: The present data highlights key logistical obstacles, including dependency on a single supplier, delays in importing materials, and high costs without market competition. In a public hospital, this vendor monopoly presents a severe financial barrier. Unlike private entities, public institutions have limited flexibility to negotiate or leverage market competition, leading to higher capital and maintenance costs that strain budgets intended for population-wide care. This risk of financial unsustainability, driven by monopolistic pricing, is a critical public health policy issue that demands governmental intervention to diversify the market or secure preferred procurement agreements.

• Infrastructure and Workforce Integration: The necessary organizational adjustments ‒ including doubling the operating room size and requiring extensive training ‒ align with literature that cites organizational and structural barriers to health technology adoption. The study's emphasis on the prolonged learning curve and the initial increase in mean total operative time (252 vs. 180.2 min) underscores the organizational resistance often encountered. This initial disruption to workflow and the demand for new competencies can lead to delays and increased initial resource consumption, a phenomenon that must be systematically managed to prevent “physician rebellion” or staff frustration, as noted in studies on health information technology implementation.

• The Productivity Paradox: The results present a nuanced trade-off: longer operative times coexisting with faster patient recovery. This aligns with the “productivity paradox” observed in health information technology, where significant investment and structural changes do not immediately translate into higher overall productivity due to learning curves and workflow adjustments. However, in the present case, the efficiency is realized postoperatively (shorter hospital stay, fewer complications, less blood loss), shifting the cost burden from bed-days (high operating cost) to the initial capital investment. For public health administrators, this means the long-term cost-effectiveness must be evaluated over the entire life cycle of the equipment, not just the procedural cost, to validate the investment.

Successfully integrating advanced surgical technologies, such as robotic systems, into a public hospital setting requires navigating a complex landscape of logistical and public health challenges. Institutions must adapt their infrastructure, retrain personnel, and manage the constraints of public funding, all while ensuring that the benefits of cutting-edge innovation are equitably distributed. This process involves not only overcoming initial operational obstacles, such as procurement hurdles, monopolistic suppliers, and budget limitations, but also strategically aligning resources to realize long-term improvements in patient outcomes and systemic efficiency. By addressing these challenges head-on, health administrators can transform the promise of technological advancement into sustainable and impactful change within the public sector.

The successful integration of robotic surgery in a university hospital depends not merely on clinical will, but on robust administrative capacity and strategic policy.

• Systemic Management: The experience demonstrates that successful adoption requires a systematic management approach using frameworks like CFIR and TDF. Meticulous resource management, continuous training, and standardized protocol adherence across multiple surgical specialties are non-negotiable for sustainability.

• Decoupling Costs from Outcomes: To ensure equity of access, governments and public health administrators must explore mechanisms to decouple the technology's clinical benefits from its prohibitive cost. This may involve incentivizing market competition, establishing shared regional robotic centers, or negotiating national agreements for consumables and maintenance.

• Continuous Evaluation: The decreasing trend in operative times illustrates a critical learning process. Continuous institutional evaluation, leveraging the collected management indicators (e.g., operative time, ICU days), is crucial for optimizing the technology’s use and ensuring its cost-effectiveness justifies its presence in a resource-limited public setting.

## Conclusion

In conclusion, while robotic surgery offers definitive clinical advantages, its implementation in the public sector presents a case study in the inherent logistical fragility of adopting expensive, singular-sourced technology. The ultimate success and ethical justification of these innovations in public health rest on developing institutional maturity that can transform a mere technological acquisition into a sustainable, well-managed system for patient care.

## Authors’ contributions

Ricardo Zugaib Abdalla: Conceptualization, methodology, investigation, data curation, formal analysis, writing - original draft, writing - review & editing.

Antônio José Rodrigues Pereira: Data curation, formal analysis (health economic analysis), writing - review & editing.

Ivan Cecconello: Project administration, conceptualization, resources, supervision, writing - review & editing.

William Carlos Nahas: Conceptualization, resources, supervision, writing - review & editing.

Evelinda Marramon Trindade: Conceptualization, methodology, data curation, formal analysis, resources, supervision, writing - review & editing.

Ana Maria Malik: Conceptualization, resources, supervision, writing - review & editing.

## Declaration of competing interest

The authors declare no conflicts of interest.

## Data Availability

The datasets generated and/or analyzed during the current study are available from the corresponding author upon reasonable request.

## Appendix Supplementary materials

Image, application 1
