# Supplementary material for: Logistic challenges in implementing a multispecialty robotic surgery program at university hospitals
Source: Clinics (Sao Paulo). 2026 Jul 16;81:101025. doi: 10.1016/j.clinsp.2026.101025 (PMC13383944; doi:10.1016/j.clinsp.2026.101025)

Supplementary Material

**Supplementary Material – Comparative Analysis of Management Indicators, detailed by Subprojects**

Comparative analysis of management results between the literature and the ICESP — in operating room use time in minutes


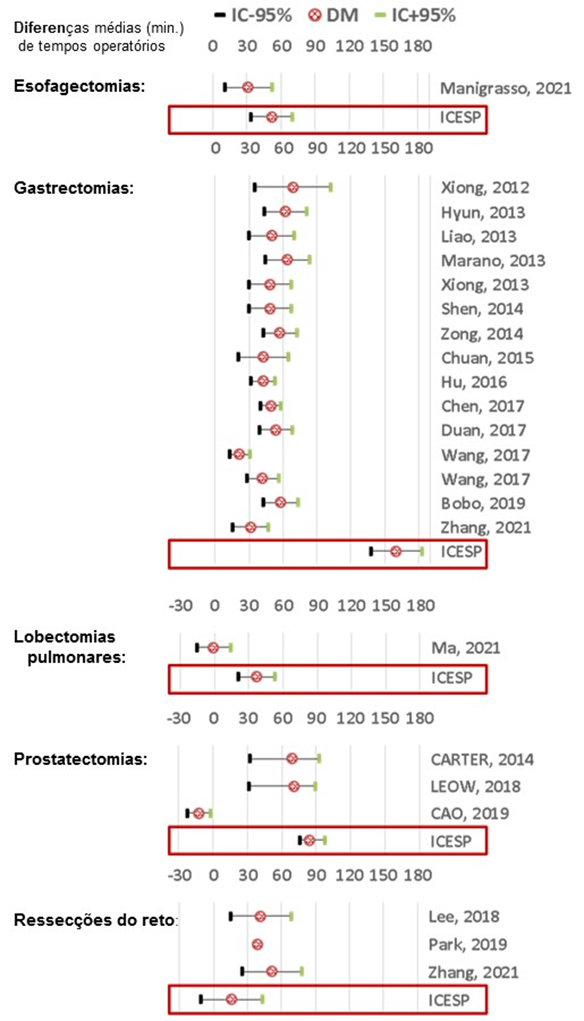


Annual evolution of the mean total operative times, paired by date of performance, in the study by subprojects and group


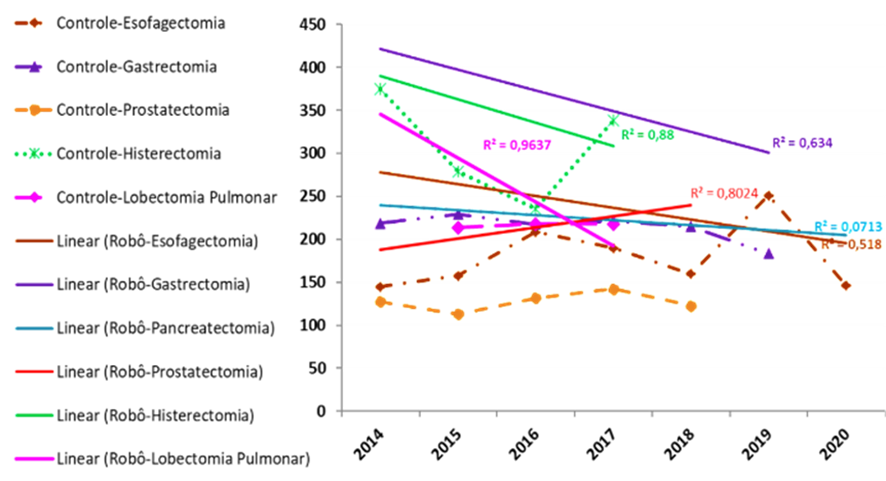


Absolute distribution of time in days of stay in the postoperative period by subprojects in the control or robotic surgery groups: means, variation and intervals with 95% confidence


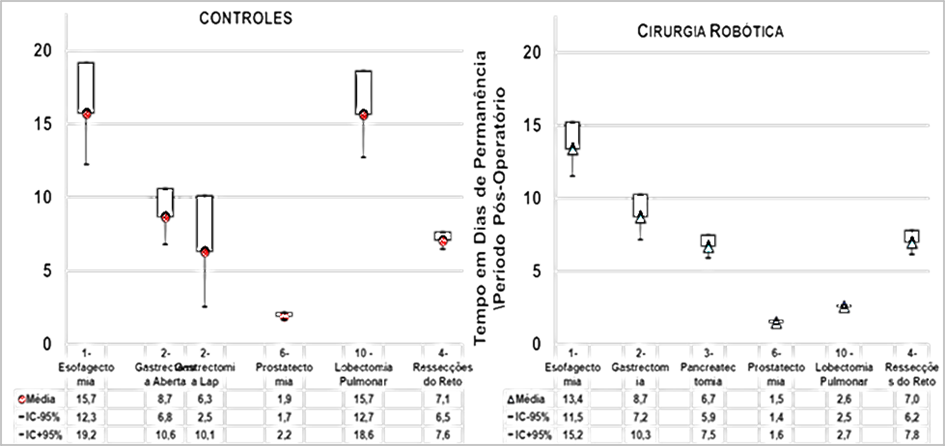


Absolute distribution of means and variability, 95% confidence intervals, of the time in days of ICU stay during the postoperative period by subprojects in the control or Robotic Surgery groups


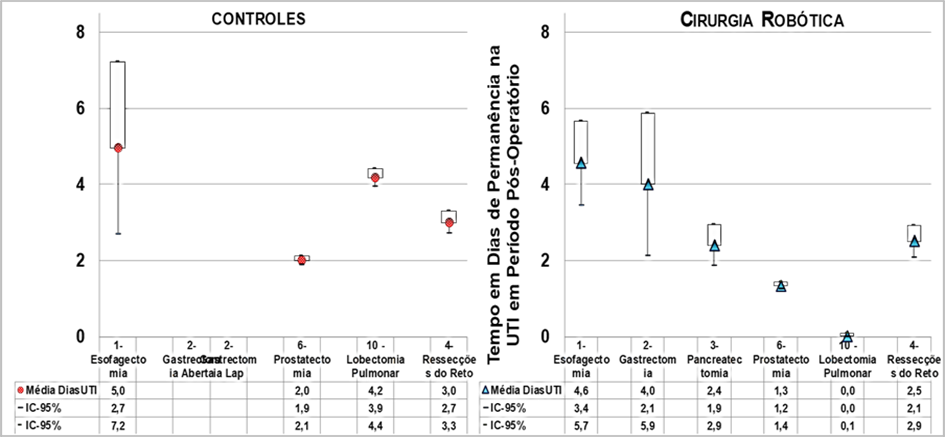

Supplement: Supplementary file 1 [file mmc1.docx]
